# Editorial: Cardiac regeneration

**DOI:** 10.3389/fcvm.2023.1178440

**Published:** 2023-03-27

**Authors:** Rajika Roy, Claudio de Lucia, Darukeshwara Joladarashi, Ajit Magadum

**Affiliations:** ^1^ Center for Translational Medicine, Lewis Katz School of Medicine, Temple University, Philadelphia, PA, United States; ^2^ Geriatric Center Frullone, ASL (Azienda Sanitaria Locale - Local Health Authority) Napoli 1 Centro, Naples, Italy

**Keywords:** cardiac regeneration, cardiomyocyte proliferation, gene therapy (GT), cell therapy, cardiac regeneration animal models, small molecule screening

**Editorial on the Research Topic** Cardiac regeneration

Cardiovascular diseases (CVD) are the leading cause of morbidity and mortality worldwide, with 19.05 million deaths in 2020. Heart failure (HF) is primarily due to the inability of the adult mammalian heart to replace the lost cardiomyocytes (CMs) following injury. Current treatments are focused on prevention rather than therapies. Therefore, developing novel therapies and finding novel molecular and cellular mechanisms are of great importance. The research topic comprises a series of original articles and reviews, and summarizes and discusses intuitive concepts, strategies, and novel inventions to regenerate or repair the injured heart.

With the focus on CM proliferation, there is a need to monitor and distinguish between cycling and non-cycling CMs. Murganti et al. described a Florescent Ubiquitination-based Cell Cycle Indicator (FUCCI) that can differentiate between cycling and non-cycling CMs and combined that with time-lapse microscopy to distinguish between cytokinesis and karyokinesis. The authors used human induced pluripotent cell (TNNT2-FUCCI)-derived CMs and screened for 94 autophagy-regulating small molecules to find a novel CM proliferator inducer. Similarly, Magadum et al. also used the FUCCI approach and developed a stable CM7/1-hgem mouse embryonic stem cell line, further differentiated into CMs to screen for novel inducers of CM proliferation. By screening the Spectrum Collection small molecule library subset, 18 potential inducers of CM proliferation were identified. Among the top four candidates were two cardiac glycosides, peruvoside and convallatoxin, and osajin and efaroxan hydrochloride. Inhibition of PTEN and GSK-3β enhanced cell cycle re-entry and progression upon stimulation with cardiac glycosides and osajin.

Myc expression has been shown to induce CM proliferation in mouse hearts when co-expressed with Cyclin T1. Boikova et al. explored the collective ability of Myc and HRas (known to stabilize Cyclin T1) in inducing CM proliferation. The overexpression of HRas*G*12*V* and constitutive Myc mutually induced adult mammalian CM proliferation but also caused CM death. Since combinatorial Cyclin T1 and Myc expression does not induce CM death in mice hearts post-MI, it could be therapeutically used. Separately, Sun et al. found cyclin-dependent kinase 9 (CDK9) an attractive therapeutic target in cardiac regeneration. CDK9 is highly expressed in the neonatal period and is usually not detected in the adult heart. CDK9 stimulates cardiac regeneration *via* serine-threonine-protein kinase 3 (SGK3) and downstream glycogen synthase kinase-3 beta (GSK-3β)/β-catenin pathway. The overexpression of CDK9 significantly stimulated mature CMs to re-enter the cell cycle and promoted cardiac repair after MI in adult mice, while CDK9 inhibition repressed CM proliferation in neonatal mice post-injury.

Duan et al. proposed metabolism (fatty acid oxidation, glycolysis, amino acid metabolism, and the tricarboxylic acid cycle), a key modulator of cardiac regeneration, to be considered as a therapeutic target. The detailed review article by Miao et al. highlighted the role of nicotinamide adenine dinucleotide phosphate oxidase (NOXs) in myocardial remodeling and its potential as a therapeutic target (Miao et al.). Amongst the different isoforms of NOX, NOX1, NOX2, NOX4, and NOX5 are found in cardiac tissue. Besides structural remodeling, NOX isoforms also play a role in modulating inflammatory responses and metabolism. In mouse models, NOX inhibitors have been shown to improve outcomes of pathological remodeling and can be further explored for clinical testing. Sierra-Pagan et al. described the role of different novel factors in cardiovascular lineage development and reprogramming that can be further exploited to better develop regenerative therapies for cardiovascular disease.

Wang et al., in a review, highlighted recent advances in cell and cell-free-based therapies for ischemic heart diseases. It is well known that the transplantation efficiency of human pluripotent stem cell (hPSC)-derived CMs (hPSC-CMs) is low because of poor cell survival in the ischemic heart. The authors listed numerous factors that regulate CM proliferation and cardiac regeneration from the last two decades.

Post-injury scar tissue lacks perfusion due to loss of vasculature. To promote re-vascularization, Spiroski et al. utilized a good manufacturing practice (GMP)-compatible human embryonic stem cell-derived endothelial cell product (hESC-ECP) that has been previously tested for its angiogenic potential. Fourteen days after cell transplantation, cardiac structure and function were better preserved. Graber et al. reported that the mechanical stimulus of shockwave therapy (SWT) could incite regenerative effects in ischemic tissue through growth factor release, modulation of the inflammatory response, and angiogenesis with functional improvement. SWT to the ischemic heart has limitations, such as the heart having a small acoustic window, restriction in accessing the treatment regions, and risk of potential lung injuries.

To study mechanistic details of *in vivo* processes, Maselli et al. established a protocol to obtain and culture 3D organotypic heart slices from porcine hearts containing intact epicardium. This approach allows cost-effective slice preparation compared to large animal testing, the ability to study an integrated group of cells instead of individual cells, and elucidating cell-ECM interactions.

In summary, the “Cardiac Regeneration” research topic addresses novel strategies and inventions, a broad explanation, and intuitive concepts on cardiac regeneration. Readers will undoubtedly appreciate the concise summaries of the contributions given in this issue that describe different ideas and strategies to induce cardiac regeneration. We hope that the ongoing efforts of investigators in cardiovascular regeneration and the published papers within this issue will one day lead to groundbreaking advances in the treatment of CVD in patients.

